# Interaction between Mitochondrial Reactive Oxygen Species, Heme Oxygenase, and Nitric Oxide Synthase Stimulates Phagocytosis in Macrophages

**DOI:** 10.3389/fmed.2017.00252

**Published:** 2018-01-22

**Authors:** Andrea Müllebner, Gabriel G. Dorighello, Andrey V. Kozlov, J. Catharina Duvigneau

**Affiliations:** ^1^Institute for Medical Biochemistry, University of Veterinary Medicine Vienna, Vienna, Austria; ^2^Ludwig Boltzmann Institute for Experimental and Clinical Traumatology, Vienna, Austria; ^3^Department of Structural and Functional Biology, Biology Institute, University of Campinas, Campinas, Brazil

**Keywords:** macrophage, phagocytosis, heme oxygenase, nitric oxide synthase, reactive oxygen species, ROS, mitochondria

## Abstract

**Background:**

Macrophages are cells of the innate immune system that populate every organ. They are required not only for defense against invading pathogens and tissue repair but also for maintenance of tissue homeostasis and iron homeostasis.

**Aim:**

The aim of this study is to understand whether heme oxygenase (HO) and nitric oxide synthase (NOS) contribute to the regulation of nicotinamide adenine dinucleotide phosphate oxidase (NOX) activity and phagocytosis, two key components of macrophage function.

**Methods:**

This study was carried out using resting J774A.1 macrophages treated with hemin or vehicle. Activity of NOS, HO, or NOX was inhibited using specific inhibitors. Reactive oxygen species (ROS) formation was determined by Amplex^®^ red assay, and phagocytosis was measured using fluorescein isothiocyanate-labeled bacteria. In addition, we analyzed the fate of the intracellular heme by using electron spin resonance.

**Results:**

We show that both enzymes NOS and HO are essential for phagocytic activity of macrophages. NOS does not directly affect phagocytosis, but stimulates NOX activity *via* nitric oxide-triggered ROS production of mitochondria. Treatment of macrophages with hemin results in intracellular accumulation of ferrous heme and an inhibition of phagocytosis. In contrast to NOS, HO products, including carbon monoxide, neither clearly affect NOX activity nor clearly affect phagocytosis, but phagocytosis is accelerated by HO-mediated degradation of heme.

**Conclusion:**

Both enzymes contribute to the bactericidal activity of macrophages independently, by controlling different pathways.

## Introduction

Macrophages are cells of the innate immune system that can be found in all tissues. They are required for maintenance of tissue homeostasis, defense against invading pathogens, tissue repair, and red blood cell recycling. Macrophages maintain homeostasis by receptor-mediated recognition and phagocytic uptake of pathogenic or microbial material and damaged or apoptotic host cells.

Degradation of the material taken up by phagocytosis is achieved *via* proteolytic enzymes and facilitated by the so-called oxidative burst. This involves the formation of reactive oxygen species (ROS) and nitric oxide (NO). Nicotinamide adenine dinucleotide phosphate (NADPH) oxidase (NOX) residing in the phagosomal membrane reduces oxygen (O_2_) to superoxide anion (${\text{O}}_{2}^{\cdot -}$). This leads to the formation of hydrogen peroxide (H_2_O_2_) and the subsequent generation of highly reactive hydroxyl radicals *via* the Fenton reaction or the synthesis of hypochlorite by myeloperoxidase. In addition, the reaction of ROS with NO yields peroxynitrite, which together with hypochlorite is very effective antimicrobial agent (1). Altogether these reactive species are termed as reactive oxygen and nitrogen species.

Also mitochondrial ROS (mtROS) plays an important role in various innate immune signaling pathways (2). They activate NOX (3–5), the NLRP3 inflammasome (6), and were shown to drive synthesis of inflammatory cytokines (7). Furthermore, mtROS are believed to increase the phagocytic activity of macrophages (8).

It is known that generation of mtROS is regulated by diatomic gaseous messengers such as NO (9) and carbon monoxide (CO) (10). NO is formed from arginine by nitric oxide synthases (NOSs) and is a ubiquitous signaling messenger involved in multiple pathophysiologic reactions (11). NO acts as a reversible inhibitor of mitochondrial respiration by competing with O_2_ for binding to the heme moiety of cytochrome *c* oxidase (COX). NO also reacts with iron sulfur clusters in complex I and II of the mitochondrial electron transport chain (mETC) (12). However, this effect is more likely assigned to the formation of peroxynitrite. Peroxynitrite inhibits the complexes of the mETC irreversibly (13). Both reversible and irreversible inhibitions of the mETC were shown to enhance mtROS formation in different model systems (9).

CO is a product of heme degradation by heme oxygenase (HO). CO targets cellular heme-containing proteins, including soluble guanylate cyclase (14, 15), NOS (16, 17), and NOX (18). Similar to NO, CO also competes with O_2_ for binding to COX. Higher levels of CO were shown to inhibit COX and to raise the production of mtROS, without decreasing mitochondrial potential (19).

Nitric oxide synthase and HO play an opposing role for the regulation of macrophage function, despite the similarity of the biological action of NO and CO. Macrophages with elevated NOS activity are considered to display a pro-inflammatory phenotype, associated with the generation of NO and peroxynitrite (20). In contrast, upregulated HO is associated with a tissue-protective phenotype (21, 22) and suppressed pro-inflammatory cytokine production. Obviously macrophages need to adapt their phenotype in accordance to the environment. Meanwhile it has become evident that the phenotype of macrophages displays a higher plasticity and a more dynamic functional repertoire than previously recognized (23).

Macrophages are particularly challenged under conditions of hemolysis, when they encounter increased levels of hemoglobin or free heme. Endocytosis of haptoglobin-bound hemoglobin *via* CD163 or uptake of the heme/hemopexin complex (24) lead to increased levels of intracellular heme (25) and initiate heme-mediated signaling cascades, among others upregulation of HO (26, 27). Recently, it was shown that the treatment of macrophages with hemin (ferric heme) inhibited phagocytosis (28). Currently, it is not clear whether the redox state of the central iron ion of the heme molecule is relevant for the inhibition of phagocytosis and thus for the regulation of macrophage function.

The iron ion in protoporphyrin can exist in a ferrous (Fe^2+^, ferroprotoporphyrin, or heme) or in a ferric form (Fe^3+^, ferriprotoporphyrin, or hemin), which can be further oxidized yielding ferryl species (29). The intracellular reactions mediated by either species are supposed to differ considerably. It is known that at least some heme-dependent processes critically depend on the redox state of heme. Ferrous heme acts as a potent catalyst of the Fenton reaction generating highly active ROS. However, as a regulator of protein function, heme appears to preferentially act in its ferric form within the cell. Ferric heme, and not ferrous heme, was shown to activate RNA-binding protein DGCR8 (30). Function of the heme-containing enzymes, NOS and cytochrome p450, requires binding of ferric heme (31). It further appears that an enhanced degradation of heme also requires ferric heme, as it mediates proteasomal degradation of the Bach repressor (32), which is required to induce HO-1 transcription. To shed light on the intracellular heme pool, we questioned whether the treatment of J774A.1 cells with ferric heme (hemin) would result in determinable amounts of ferrous heme.

### Aims

The aim of this study is to investigate the contribution of NOS and HO to the regulation of NOX activity and phagocytosis. Particular attention was paid to the role of mtROS and hemin as modulators of ROS generation and phagocytic activity.

## Materials and Methods

### Material

All chemicals used in this study were obtained from Sigma-Aldrich (St. Louis, MO, USA). HO inhibitors [tin protoporphyrin (SnPP), zinc protoporphyrin (ZnPP), and chromium mesoporphyrin (CrMP)] were obtained from Frontier Scientific (Logan, UT, USA). Fetal calf serum was purchased from Bio & Sell (Nürnberg, Germany).

### Cell Culture

J774A.1 mouse macrophages (TIB-67™; ATCC^®^, Manassas, VA, USA) were grown in Dulbecco’s modified Eagle’s medium containing high glucose (glucose 25 mM, glutamine 4 mM, sodium bicarbonate 1.5 g/l, and sodium pyruvate 1 mM), supplemented with 10% fetal calf serum. They were grown either adherently in cell culture flasks or in suspension in roller culture. The cells were kept in a humid incubator with 95% of air and 5% of carbon dioxide at 37°C.

### Inhibition of Underlying HO Activity of J774A.1 Cells

The presence of basal HO activity was confirmed in homogenates of J774A.1 cells using an optimized enzyme-coupled spectrophotometric assay detecting the final product bilirubin (33) (data not shown). Full inhibition of enzyme activity determined in the presence of hemin (20 µM) was found for the HO inhibitors ZnPP, SnPP, and CrMP at equimolar concentration (20 µM; data not shown). *In situ* HO activity was confirmed by the detection of bilirubin in the cell culture supernatant formed from hemin (20 µM) after incubation overnight as elsewhere described (33).

### Inhibition of NOS Activity

The basal activity of NOS was confirmed indirectly by the acceleration of mitochondrial O_2_ consumption rates by NOS inhibitors (data not shown). Effective concentrations of the inhibitors were determined by measuring nitrite (NO_2_^−^) formation from lipopolysaccharide-treated (1 µg/ml for 16 h) J774A.1 cells. NO_2_^−^ formation was fully inhibited at concentrations of 10 mM l-N^G^-Nitro-arginine methyl ester (l-NAME) and 50 μM S-methyl-thiocitrulline (TC; data not shown).

### Determination of Total ROS, mtROS, and NADPH-Derived ROS (NOX-ROS)

**Figure 1:**
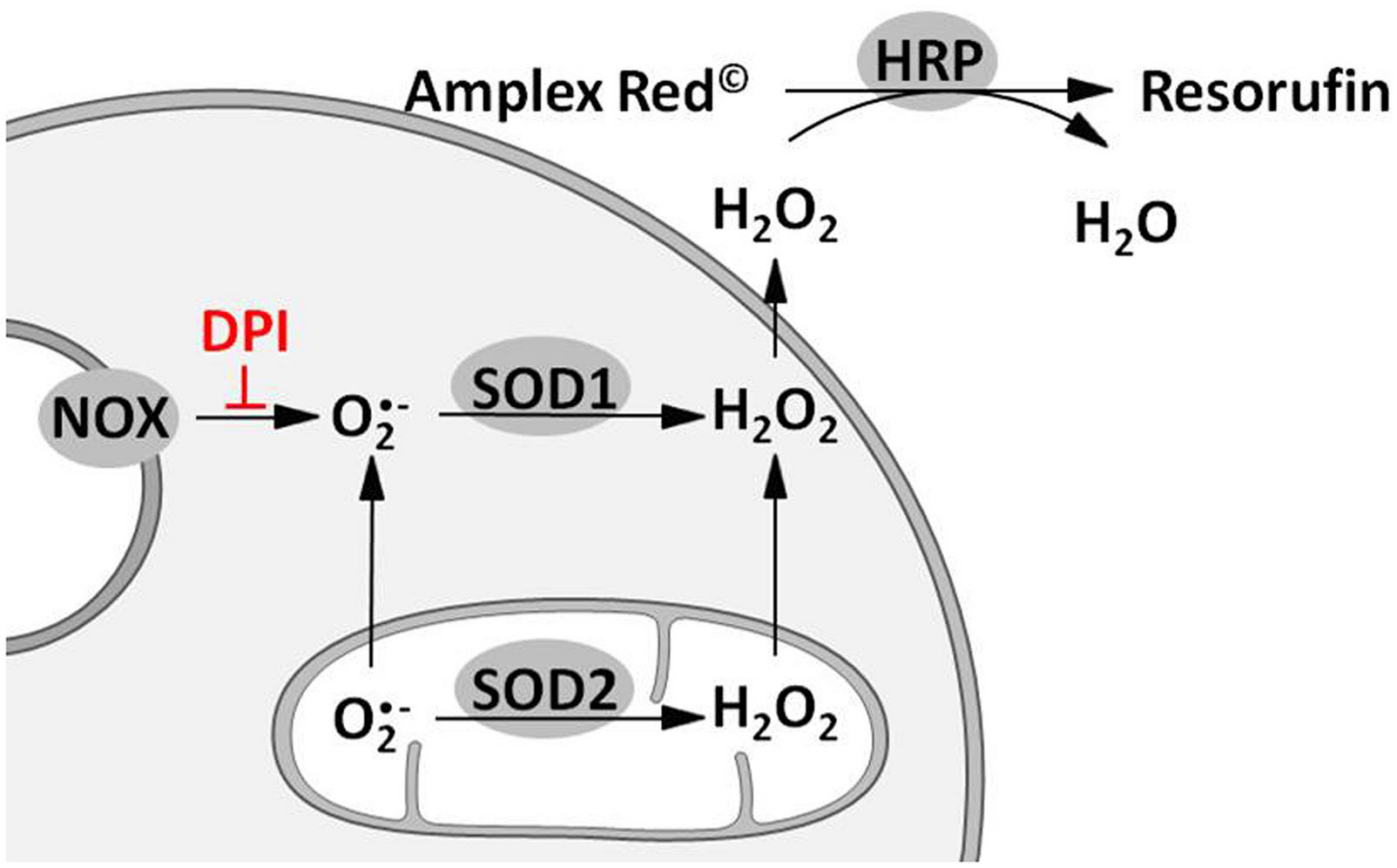
Scheme illustrating the approach for measuring total, mitochondrial (mt) and nicotinamide adenine dinucleotide phosphate oxidase (NOX)-derived reactive oxygen species (ROS) formation. Superoxide radicals formed by either mitochondrial electron transport chain or NOX are converted to hydrogen peroxide (H_2_O_2_) by mitochondrial and cytoplasmic superoxide dismutase (SOD1 and SOD2). H_2_O_2_ capable of diffusing through membranes reacts with Amplex^®^ red to resorufin on an equimolar basis in presence of horseradish peroxidase (HRP). Hence fluorescence of resorufin (excitation: 530–560 nm; emission: 590 nm) can be used as an indirect measure of ROS formation. Determination of mitochondrial ROS (mtROS) is achieved by inhibition of NOX using diphenyleneiodonium chloride (DPI). Difference of total and mtROS formation reflects NOX-ROS levels.

Reactive oxygen species formation was quantified using Amplex^®^ red (Molecular Probes, Eugene, Oregon, USA). Before the experiment (24 h), the cells were seeded in low-fluorescence 96-well plates at a density of 6 × 10^4^ cells per well. Experiments were performed in 100 µl Krebs buffer [NaCl (135 mM), KCl (5 mM), MgSO_4_ (1 mM), K_2_HPO_4_ (0.4 mM), CaCl_2_ (1 mM), HEPES (15 mM), and glucose (25 mM); pH 7.4] containing Amplex^®^ red (10 µM), horse radish peroxidase (HRP; 0.2 U/mL), and phorbol 12-myristate 13-acetate (PMA; 1 µM). Contribution of mitochondria, NOS, and HO to the determined ROS formation was investigated using mitoTEMPO (0.5 µM) or inhibitors of either NOS (L-NAME, 10 mM) or HO (SnPP, 20 µM). Each condition was measured in the presence and absence of the NOX inhibitor diphenyleneiodonium chloride (DPI, 0.05 µM). Plates were kept in the dark at 37°C for 35 min measuring fluorescence of the formed resorufin (excitation: 530 nm, emission: 590 nm) every 5 min. The fluorescence signals were corrected for background fluorescence determined in wells without cells. The slope of the regression lines calculated from the first 15 min of resorufin formation was taken as H_2_O_2_ release rate and was displayed as percentage of total H_2_O_2_ release rate in controls. Rates detected in the absence of DPI reflected total H_2_O_2_ production; rates in the presence of DPI were taken as a measure for the mitochondrial H_2_O_2_ production. The difference between total and mitochondrial H_2_O_2_ production was used as a measure for the H_2_O_2_ generation by NOX activity (Figure 1). Modulation of ROS formation by mtROS (mitoTEMPO) was determined in *n* = 5, by NOS (L-NAME) and HO (SnPP) in *n* = 3 independent experiments. Each condition was measured in technical replicates of *n* = 6.

### Determination of Phagocytosis

Phagocytic activity was assessed by using Phagotest™ (Glycotype Bioscience; Berlin, Germany) according to the following protocol. 1 × 10^6^ cells were suspended in 200 µl Dulbecco’s modified Eagle’s medium buffered with 25 mM HEPES. The suspension was treated for 60 min with vehicle (dimethyl sulfoxide) or hemin (20 µM) with or without inhibitors of NOS (L-NAME, 10 mM; TC, 50 µM) or HO (ZnPP or CrMP, 20 µM). The viability of the cells after the initial 60 min of treatment (vehicle, hemin NOS/HO inhibitors) was analyzed by flow cytometry after propidium iodide staining (1 µg/ml) and was always higher than 90% (data not shown). Fluorescein isothiocyanate-labeled bacteria (20 µl diluted 1:2) were added, and the suspension was incubated at 37°C under shaking. After 30 min, phagocytosis was stopped by placing tubes on ice. Each sample was analyzed in duplicates. In addition, a control sample for each condition was kept on ice to prevent phagocytosis. After adding of 100 µl ice-cold quenching solution and incubation on ice for 3 min, cells were washed with ice-cold wash buffer (phosphate-buffered saline containing 0.3% fetal calf serum), fixed with 1% paraformaldehyde for 20 min at room temperature, washed again, and resuspended in 200 µl wash buffer. Uptake of bacteria was assessed on a FACScan (Becton Dickinson, Franklin Lakes, NJ, USA) flow cytometer and analyzed using Cell Quest 3.1 software (Becton Dickinson, Franklin Lakes, NJ, USA). A live gate was set in the scatter plot (forward scatter versus side scatter) to exclude debris. The green fluorescence histogram (FL1, 530/30) was analyzed. Autofluorescence of J774A.1 cells was assessed in the respective control samples to set a marker for discrimination between non-phagocytosing and phagocytosing cells. Percentage of gated events above this marker equals the population of phagocytosing cells in the samples. The mean fluorescence in this population was taken as a measure for the number of bacteria taken up per individual cell. For each condition, *n* = 4 independent experiments were performed.

### Determination of Ferrous Heme by Electron Spin Resonance (ESR) Spectroscopy

To determine the occurrence of intracellular ferrous heme, we analyzed the spectra of suspension cells treated for 24 h with vehicle, hemin, or hemin plus diethylenetriamine NONOate (DETA/NO). 5 min before collecting the cells or the supernatants, 1 mM diethylamine NONOate (DEA/NO; 1 mM) was added to convert all free ferrous heme into the nitrosylated form. The cell suspension was centrifuged (350 × g), the supernatant was harvested, and the pelleted cells were washed twice with PBS and resuspended in 300 µl phosphate-buffered saline. 300 µl of the samples were aspirated in standard 1 ml syringes and shock frozen in liquid nitrogen. ESR spectra were recorded at liquid nitrogen temperature (−196°C) with a Magnettech MiniScope MS 200 ESR spectrometer (Magnettech Ltd., Berlin, Germany). The general settings were as follows: modulation frequency, 100 kHz; microwave frequency, 9.425 GHz; microwave power, 8.3 mW; modulation amplitude, 5 G; gain, 200; range, 330 ± 20 mT. Two independent experiments were performed.

### Data Analysis and Statistics

Data processing and visualization were performed using Excel or GraphPad Prism v6.01 (GraphPad Software Inc., La Jolla, CA, USA). Data are presented as means ± SEM. Differences between groups were assessed using paired *t*-test for ROS formation and for phagocytosis using matched two-way ANOVA followed by Sidak’s multiple comparisons using GraphPad Prism. Differences were considered significant when *p* < 0.05.

## Results

### Determination of Total ROS, mtROS, and NOX-ROS

**Figure 2:**
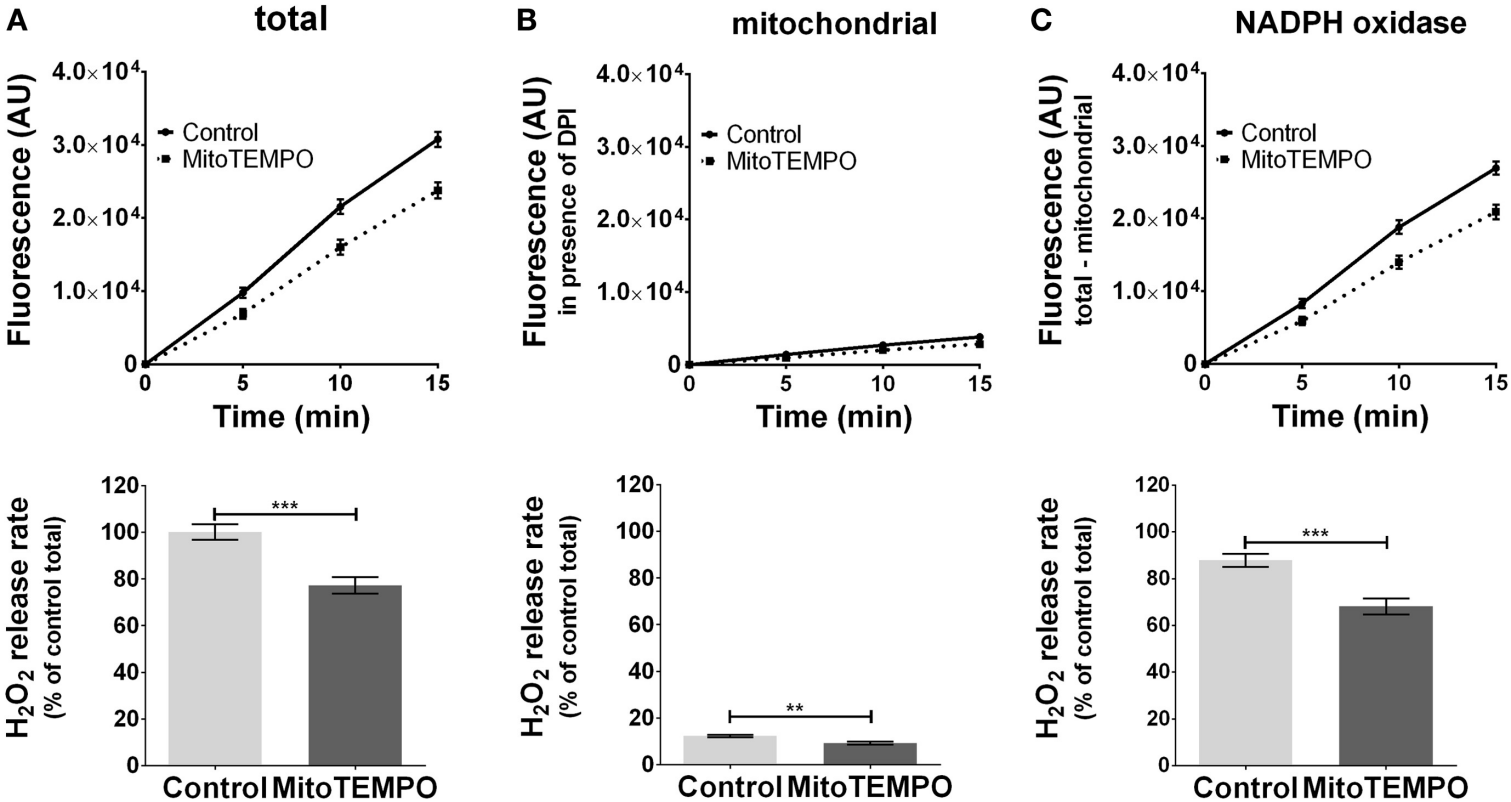
Modulation of reactive oxygen species (ROS) formation in J774A.1 cells by mitochondria-targeted antioxidant MitoTEMPO. Hydrogen peroxide (H_2_O_2_) formation in J774A.1 cells treated with dimethyl sulfoxide (control) or MitoTEMPO (0.5 µM) was triggered by phorbol 12-myristate 13-acetate (1 µM). Formation of resorufin from Amplex^®^ red was assessed over 15 min. H_2_O_2_ release rates correspond to slopes calculated from resorufin formation kinetics and are displayed as percentage of total H_2_O_2_ release rate of the controls **(A)**. Mitochondrial H_2_O_2_ release **(B)** was assessed by blocking nicotinamide adenine dinucleotide phosphate oxidase (NOX) using diphenyleneiodonium chloride (DPI; 0.05 µM). NOX H_2_O_2_ release **(C)** equals the difference of mitochondrial **(B)** from total H_2_O_2_ release **(A)**. Data are presented as mean ± SEM of five independent experiments. Significant differences (*p* < 0.05) are indicated (**p < 0.01 and ***p < 0.005).

The ROS formation from unstimulated cells was below the detection limit (data not shown). The application of PMA triggered ROS generation (Figure 2A) by activating both mitochondria- (Figure 2B) and NOX-dependent (Figure 2C) ROS formation. On treatment with the mitochondria-targeted antioxidant mitoTEMPO, total ROS formation was reduced by 30% (Figure 2A). MitoTEMPO at 0.5 µM was sufficient to scavenge about 25% of mtROS, corresponding to a portion of less than 5% of total ROS (Figure 2B). This in turn substantially reduced NOX-ROS formation (Figure 2C).

### Effect of NOS Inhibition on Formation of mtROS and NOX-ROS

**Figure 3:**
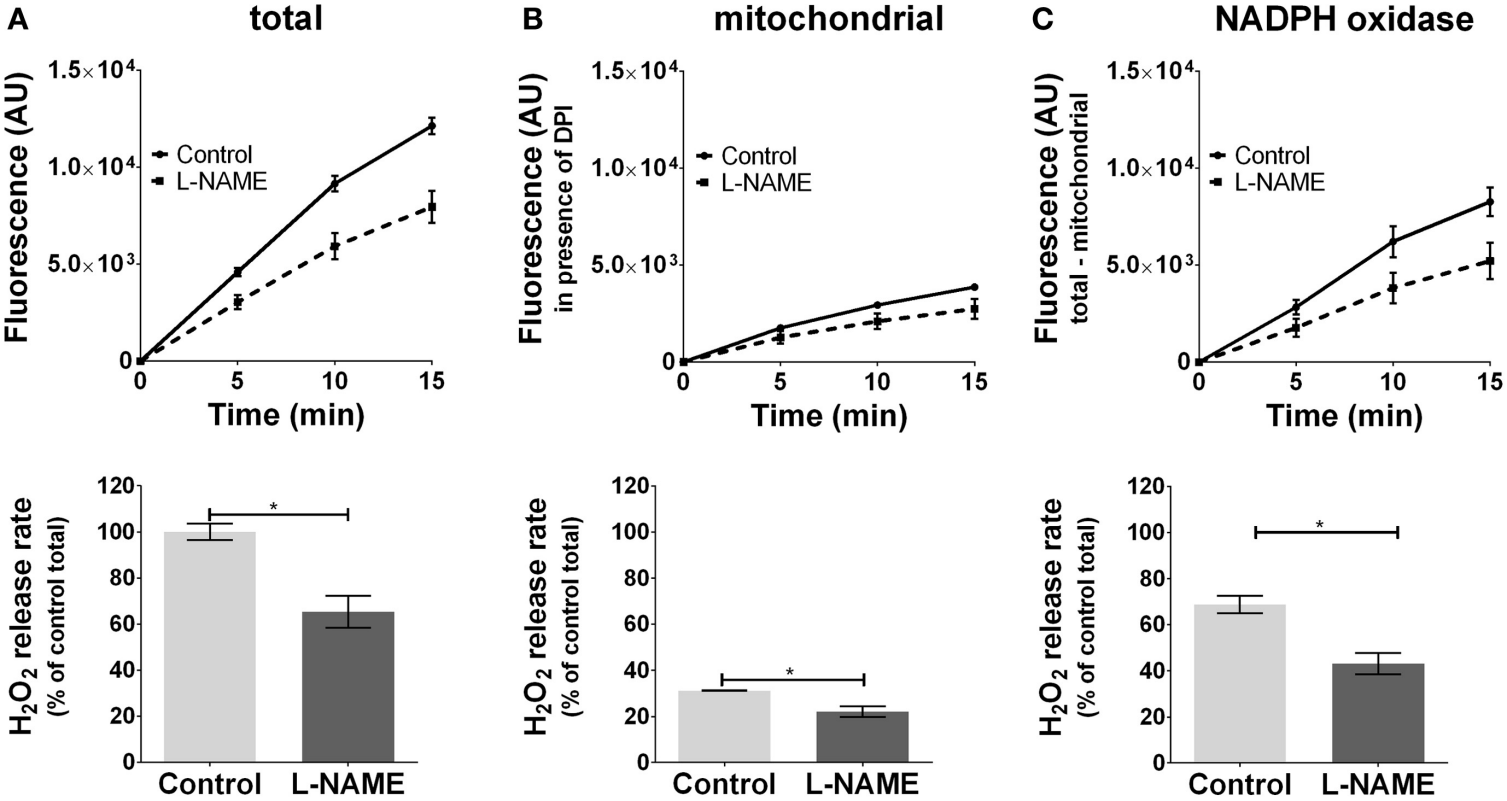
Modulation of reactive oxygen species formation in J774A.1 cells by inhibition of nitric oxide synthase activity with l-N^G^-nitro-arginine methyl ester (L-NAME). Hydrogen peroxide (H_2_O_2_) formation in J774A.1 cells in the absence (control) or presence of l-NAME (10 mM) was triggered by phorbol 12-myristate 13-acetate (1 µM). Formation of resorufin from Amplex^®^ red was assessed over 15 min. H_2_O_2_ release rates correspond to slopes calculated from resorufin formation kinetics and are displayed as percentage of total H_2_O_2_ release rate of the controls **(A)**. Mitochondrial H_2_O_2_ release **(B)** was assessed by blocking nicotinamide adenine dinucleotide phosphate oxidase (NOX) using diphenyleneiodonium chloride (DPI; 0.05 µM). NOX H_2_O_2_ release **(C)** equals the difference of mitochondrial **(B)** from total H_2_O_2_ release **(A)**. Data are presented as mean ± SEM of three independent experiments. Significant differences (*p* < 0.05) are indicated (*p < 0.05).

Inhibition of basal NOS activity reduced ROS formation by 30% (Figure 3A) in J774.A1 cells. NOS inhibitor attenuated mtROS formation (Figure 3B), which was accompanied by decreased ROS formation by NOX (Figure 3C) showing that NOS contributes to an enhanced NOX-ROS generation. These data suggest that NO activates NOX in a mtROS-dependent manner.

### Effect of HO Inhibition on Formation of mtROS and NOX-ROS

**Figure 4:**
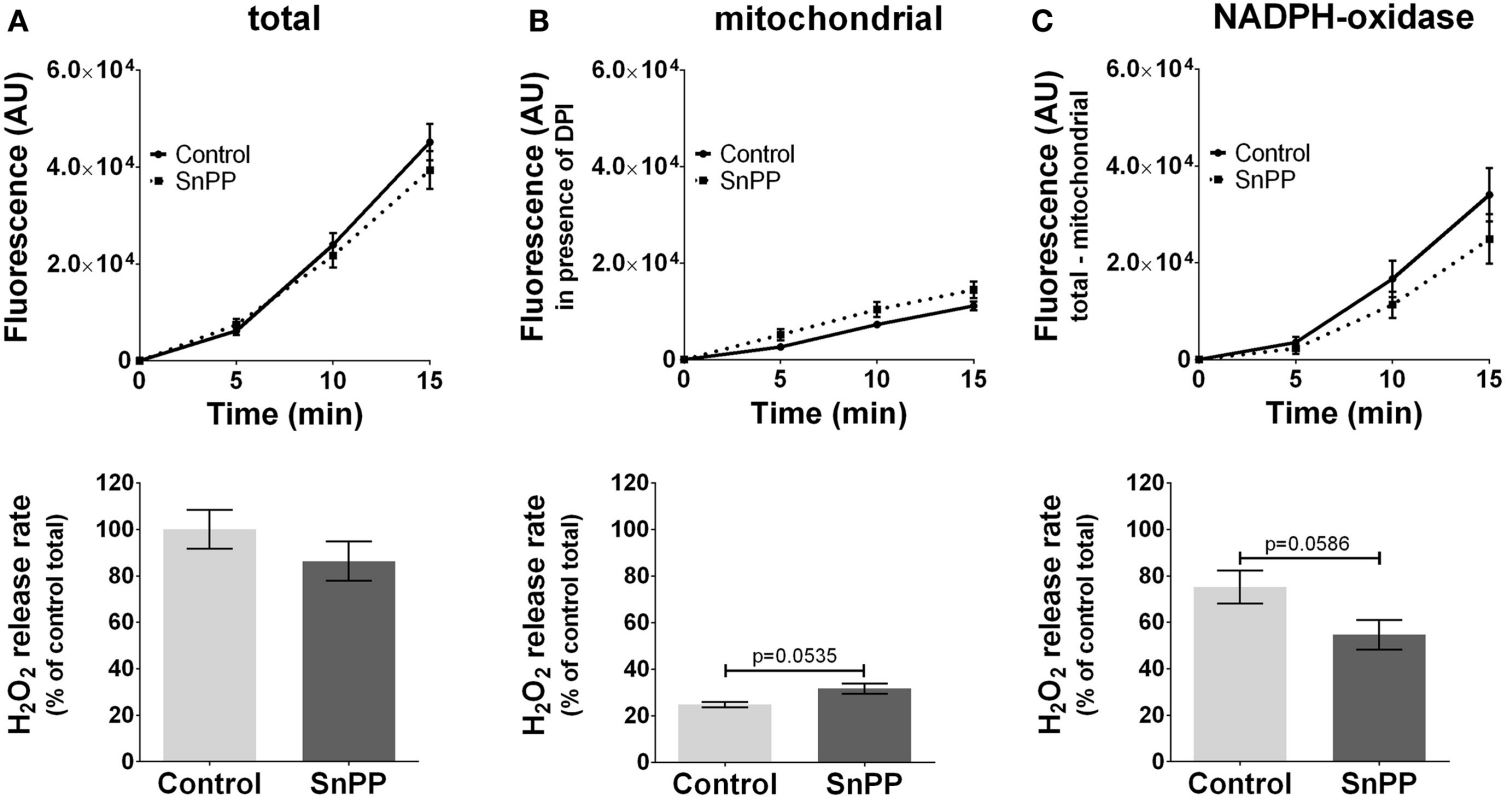
Modulation of reactive oxygen species formation in J774A.1 cells by inhibition of heme oxygenase activity with tin protoporphyrin (SnPP). Hydrogen peroxide (H_2_O_2_) formation in J774A.1 cells treated with dimethyl sulfoxide (control) or with SnPP (20 mM) was triggered by phorbol 12-myristate 13-acetate (1 µM). Formation of resorufin from Amplex^®^ red was assessed over 15 min. H_2_O_2_ release rates correspond to slopes calculated from resorufin formation kinetics and are displayed as percentage of total H_2_O_2_ release rates of the controls **(A)**. Mitochondrial H_2_O_2_ release **(B)** was assessed by blocking nicotinamide adenine dinucleotide phosphate oxidase (NOX) using diphenyleneiodonium chloride (DPI; 0.05 µM). NOX H_2_O_2_ release **(C)** equals the difference of mitochondrial **(B)** from total H_2_O_2_ release **(A)**. Data are presented as mean ± SEM of three independent experiments. *p* values obtained from the statistical analyses are indicated.

In contrast to inhibitors of NOS, we did not observe a significant effect of HO inhibitors neither on total ROS (Figure 4A) nor on mtROS (Figure 4B) nor on NOX-ROS (Figure 4C) formation. However, there was a high tendency of an increased mtROS formation from mitochondria (*p* = 0.0535) when HO was inhibited. Simultaneously the inhibition of HO resulted in a decreased NOX-ROS formation by trend (*p* = 0.0586). Although the determined effects of HO inhibition were not strong enough to result in significant differences using a replicate number of 3, we cannot completely exclude a possible contribution of HO to ROS production. Thus, our data suggest that HO and NOS may affect mitochondrial ROS production in an opposite way.

### Effect of NOS Inhibitors on Phagocytosis

**Figure 5:**
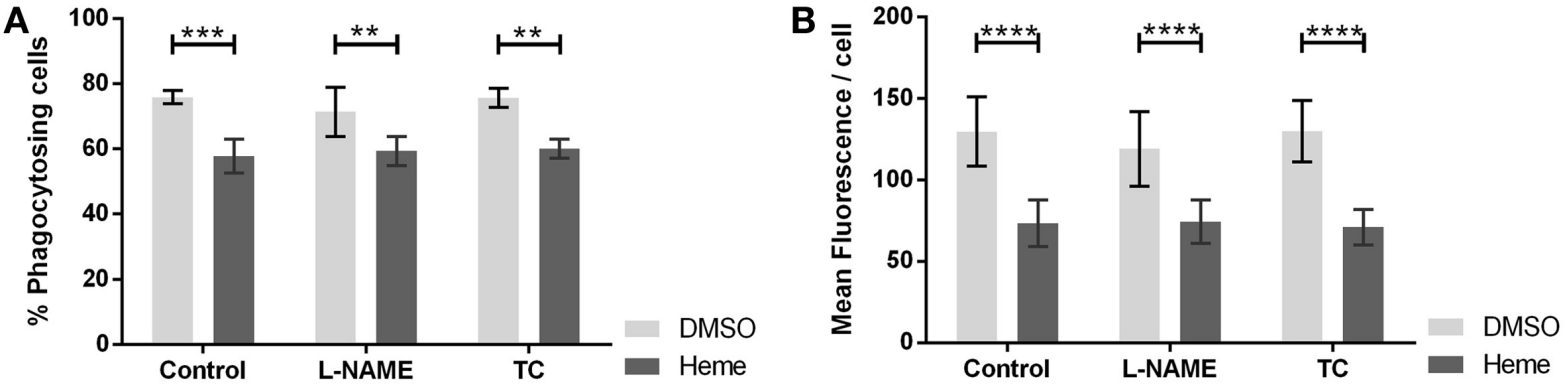
Effect of nitric oxide synthase (NOS) inhibition on phagocytic activity of J774A.1 cells. The amount of phagocytosing cells from the entire population [in % **(A)**] and average amount of phagocytosed bacteria per cell **(B)** was determined. Macrophages were treated with dimethyl sulfoxide or hemin (20 µM) alone (control) or with additional NOS inhibitors l-N^G^-nitro-arginine methyl ester (L-NAME; 10 mM) or *S*-methyl-l-thiocitrulline (TC; 50 µM). Phagocytic activity was assessed using Phagotest Kit (Glycotype Technology). Data are presented as mean ± SEM of four independent experiments. Significant differences are indicated as follows (**p < 0.01, ***p < 0.005, and ****p < 0.001).

**Figure 6:**
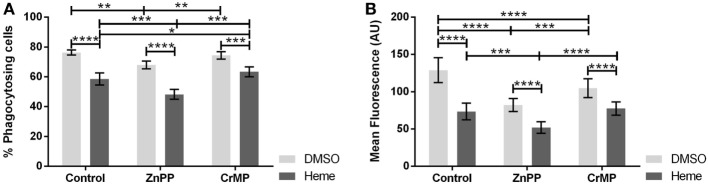
Effect of heme oxygenase (HO) inhibition on phagocytic activity of J774A.1 cells. The amount of phagocytosing cells from the entire population [in % **(A)**] and average amount of phagocytosed bacteria per cell **(B)** was determined. Macrophages were treated with dimethyl sulfoxide (DMSO) or hemin (20 µM) alone (control) or with additional HO inhibitors zinc protoporphyrin (ZnPP; 20 µM) or chromium mesoporphyrin (CrMP; 20 µM). Phagocytic activity was assessed using Phagotest Kit (Glycotype Technology). Data are presented as mean ± SEM of four independent experiments. Significant differences are indicated as follows (*p < 0.05, **p < 0.01, ***p < 0.005, and ****p < 0.001).

Inhibition of NOS with l-NAME altered neither the percentage of phagocytosing cells (Figure 5A) nor the average number of bacteria ingested per cell (Figure 5B). We confirmed the results using TC, an alternate inhibitor of NOS. Considering that the action of HO and NOS at the level of phagocytosis can be synergistic, we repeated the experiment in the presence of hemin, the substrate of HO. Hemin strongly impaired phagocytic activity by reducing the number of phagocytosing cells (Figures 5A and 6A) and even more the amount of bacteria ingested per cell (Figures 5B and 6B). But even in the presence of hemin, NOS inhibitors had no effect on the rate of phagocytosis (Figures 5A,B).

### Effect of HO Inhibitors on Hemin Modulated Phagocytosis

Since an inhibition of phagocytosis could be exerted either by hemin or by the products of HO reaction, we questioned whether the inhibition of basal HO activity would prevent the observed phagocytosis inhibition. Therefore, we studied phagocytosis in the presence of two different HO inhibitors, ZnPP and CrMP. Both HO inhibitors *per se* reduced phagocytosis (Figures 6A,B), especially the number of ingested bacteria per cell (Figure 6B), suggesting that the inhibition of phagocytosis is due to the accumulation of heme. This was confirmed by the further inhibition of phagocytic activity in presence of hemin (Figures 6A,B).

### Occurrence of Ferrous Heme within Cells or Cell Supernatant

**Figure 7:**
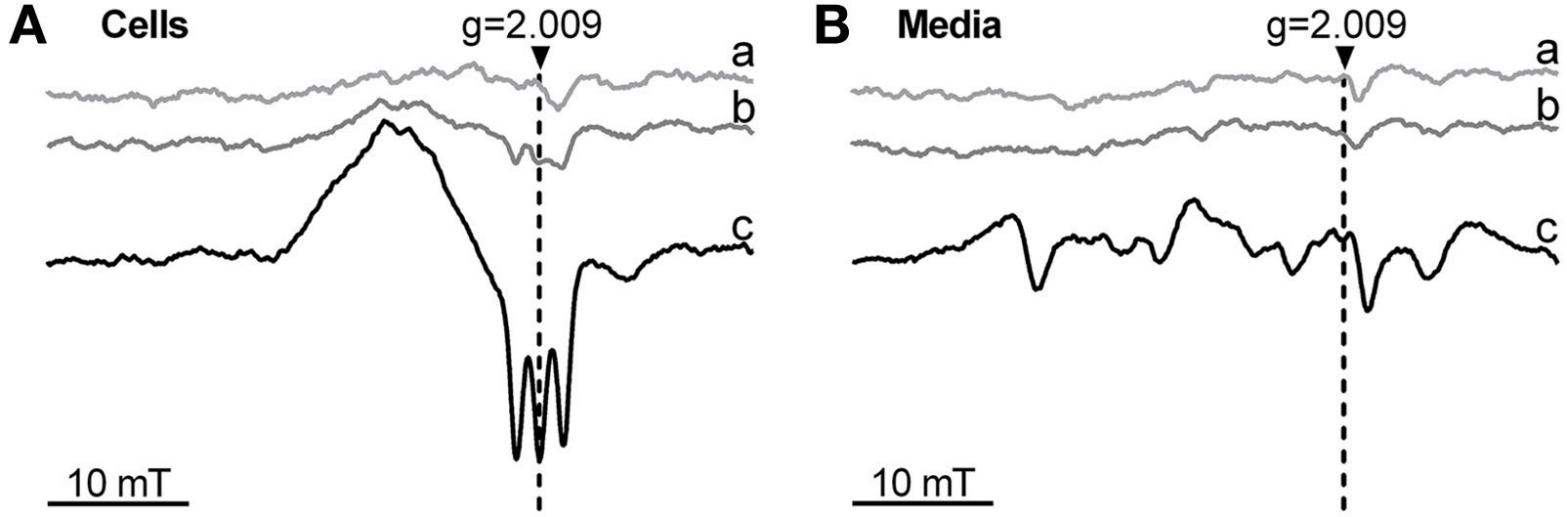
Occurrence of ferrous heme determined by low-temperature electron paramagnetic resonance (ESR) spectra of J774A.1 cells **(A)** and incubation media **(B)** after treatment with hemin and nitric oxide (NO) donors. Cells were incubated for 24 h with medium alone (control; a) or in the presence of hemin (20 µM; b) or hemin (20 µM) and diethylenetriamine NONOate (100 µM; c). After incubation, cells were treated with diethylamine NONOate (1 mM) for 5 min. Released nitric oxide and ferrous heme form a nitrosyl-heme complex giving a triplet structured signal centered at g = 2.009. ESR parameters are described in Section “Materials and Methods.” Representative spectra of two independent experiments are shown. g, g-factor; mT, millitesla.

Phagocytosis was modulated by changes of HO activity, which affects the level of the intracellular heme pool. It is possible that the redox state of heme plays a role in this regulation. To understand whether treatment of the cells with ferric heme (hemin) would result in occurrence of intracellular ferrous heme, we additionally treated the cells with the NO-donor DEA/NO, which allows the detection of ferrous heme in its nitrosylated form by low-temperature ESR, due to its unique spectrum. Cells exposed to 1 mM NO-donor DEA/NO for 5 min did not display any NO-related signal (Figure 7A). Cells treated with hemin for 24 h and exposed to 1 mM NO-donor DEA/NO for the final 5 min showed the ESR signal typical for NO-heme centered at g = 2.009 and with the characteristic triplet splitting (Figure 7A). This signal indicates that at least a small portion of intracellular heme–iron occurs in a ferrous form. The signal was strongly increased after the simultaneous treatment with hemin and DETA/NO for 24 h (Figure 7A) and exposure to 1 mM NO-donor DEA/NO for the final 5 min, suggesting the accumulation of nitrosylated heme inside the cell. None of the supernatants showed a signal arising from nitrosyl–heme complexes. This indicates that exclusively ferric heme is present extracellularly in the supernatants (Figure 7Ba–c) and that the reduction step required to form ferrous from ferric heme, allowing the formation of nitrosyl–heme complexes, occurred inside the cells.

## Discussion

We studied the role of HO and NOS on ROS generation and phagocytosis, two major tasks of macrophages. NOS and HO play an opposing role in the regulation of macrophages despite the similarity of the biological action of their products NO and CO. Macrophages with elevated NOS activity are considered to display a pro-inflammatory phenotype, which is associated with the generation of NO and peroxynitrite (20). In contrast, upregulated HO is associated with a tissue-protective phenotype and a suppressed pro-inflammatory cytokine production. The anti-inflammatory and tissue-protective effects are believed to be mediated mainly by the HO reaction products, CO (34, 35) and bilirubin (36). In addition, HO products were shown to inhibit inducible NOS expression and NO formation (37, 38). Macrophages activated by pro-inflammatory stimuli produce high levels of NO, by far exceeding the levels of CO that can be reached by active HO. We found that NOS product formation in LPS-stimulated J774A.1 cells was several 100-fold higher than the generation of HO products upon hemin stimulation within the same time (data not shown). Both NO and CO compete with oxygen for the binding to heme proteins of the mETC, such as COX. Therefore, the different binding affinities and the concentration ratios of all three ligands to each other determine the overall effects on the mETC. To avoid competition by excessively produced NO, we used macrophages without previous activation (resting macrophages), which display only basal NOS activity. To trigger ROS generation or phagocytosis, we used PMA stimulation or bacterial preparations, respectively.

### mtROS Amplify NOX-ROS Formation

In J774A.1 cells, PMA induced ROS generation from both mitochondria and NOX. This is in line with the former report using human lymphoblasts (39). It was previously shown that mtROS may enhance the activity of NOX (3–5). Since we have found that the amount of ROS generated by mitochondria was about 10 times lower compared to that generated from NOX, we tested whether these levels would be sufficient for an enhancement of NOX activity in J774A1 cells, using the mitochondria-targeted ROS scavenger mitoTEMPO. As expected, mitoTEMPO attenuated mtROS generation, and in addition, it also attenuated generation of NOX-ROS, confirming previous findings in activated macrophages (5). This shows that mtROS contribute to NOX-ROS generation also in resting macrophages. In addition, these data show that mtROS operate as an amplifier of the NOX-ROS generation and underpin the role of mtROS as an essential regulator of the bactericidal activity of macrophages (5).

Formation of mtROS was shown to be modulated by NO and CO (9, 40). Therefore, both gaseous messengers are possible candidates for an indirect modulation of NOX-ROS generation in macrophages. In addition, it was suggested that NOX as a heme protein may be directly inhibited by NO or CO (41, 42). In resting macrophages, NOS- and HO-derived NO and CO are endogenously produced at low levels. To elucidate the potential of such basal enzyme activity to influence the activity of NOX *via* formed gaseous messengers, we applied specific NOS and HO inhibitors.

### Basal NOS Contributes to NOX-ROS Formation *Via* mtROS in Resting Macrophages

Inhibition of NOS decreased mtROS formation and subsequently NOX-ROS generation. These findings confirm that the ROS-NOS cycle, which was previously described in hepatocytes (7), also exists in macrophages. In addition, the decrease of formation of NOX-ROS by NOS inhibition shows that mtROS formation is modulated by NO, possibly *via* inhibiting mETC. It should be noted that the macrophages were not activated and that NO production under these conditions is rather low. However, our data suggest that also in resting macrophages basal NOS activity is sufficient to activate NADPH oxidase in a mtROS-dependent manner.

### No or Limited Contribution of Basal HO to ROS Formation in Resting Macrophages

While NOS inhibition affects only NO levels, HO inhibition not only leads to decreased CO levels but also simultaneously to decreased levels of ferrous iron and bilirubin. In addition, the inhibition of HO leads to the accumulation of intracellular heme and may thereby promote other heme-mediated reactions as shown by others (43). Therefore, it is not possible to attribute effects in the HO inhibitor-treated cells solely to the absence of a single HO product. This and the comparably low concentrations of HO products formed by basal HO activity might explain that in our model ROS generation was not effected upon inhibition of HO, contrasting previous findings in RAW 264.A cells treated with HO-derived products (35, 36). However, there was a trend for a reduction of NOX-ROS generation when HO was inhibited. We found additionally that HO inhibition increased the mtROS formation by trend, which is in contrast to the findings obtained for the inhibition of NOS. Due to the limited number of replicates (*n* = 3), it is not possible to clearly exclude a modulatory effect of HO on NOX-ROS and mitochondrial ROS production and a possible contribution of HO on ROS generation in macrophages. Anyway, our data show that HO and NOS differ considerably regarding their contribution to mitochondrial ROS production. To find out whether these effects are of biological relevance, further studies have to be performed.

### NOS Does Not Affect Phagocytosis in Resting J774A.1 Cells

An efficient phagocytosis is essential for macrophage bactericidal function, besides sufficient ROS formation. In pro-inflammatory macrophages, both NOS (44) and HO activities (45, 46) accelerate the process of phagocytosis. On the other hand, it was shown that knocking out HO-1 results in an increased expression of macrophage-specific scavenger receptor A (47), which is required for phagocytosis and inflammatory signal release (48). Therefore, we questioned whether NOS and HO activity contribute to the phagocytic activity in resting macrophages as well. NOS inhibitors did neither influence the number of phagocytosing cells nor influence the amount of phagocytosed bacteria. It is known that the substrate of HO, hemin, is an inhibitor of phagocytosis in macrophages (28). Since it is not clear whether NOS or HO products show additional modulating properties, we investigated phagocytosis in cells treated with hemin and simultaneously inhibited HO and NOS activity.

### Hemin Impairs Phagocytosis in Resting J774A.1 Cells

We observed that hemin reduced phagocytic activity, which is in line with the study performed by Martins et al. (28). Both the number of phagocytosing cells and the amount of bacteria phagocytosed per cell were decreased by hemin treatment, and thus, we questioned whether this effect is mediated by the formation of HO products or by heme itself.

### HO Partly Restores Phagocytosis by Degradation of Heme

Co-treatment of cells with hemin and an HO inhibitor further decreased the phagocytic activity, suggesting that hemin itself, rather than the HO products, modulate phagocytic activity. Since active HO degrades heme, these findings suggest that HO contributes indirectly to the efficiency of phagocytosis. This is supported by the findings that macrophages and neutrophils from HO-1 knockout animals showed an impaired bacterial clearance upon treatment with heme (28). In addition, we found that treatment with HO inhibitors alone also reduced phagocytic activity. HO inhibition is supposed to increase the levels of intracellular heme pool, even in the absence of an external heme supplementation. Thus, our findings indicate that the endogenous basal HO activity of J774A.1 cells is sufficient to accelerate phagocytosis. Our findings further suggest that it is not the treatment with hemin itself, but the change of the level of the intracellular heme pool, which is responsible for the modulation of phagocytosis.

### Heme Exists as Ferrous Heme in the Intracellular Heme Pool

The reactions catalyzed by heme are dependent on its redox state. The availability of heme in the appropriate redox state appears to play an important role for its regulator function (49). For example, ferric heme, and not ferrous heme, was shown to activate RNA-binding protein DGCR8 (30). It is not known whether (i) the redox state of heme is relevant for the regulation of phagocytosis, (ii) heme within the heme pool exists predominately in its ferrous or ferric form in resting macrophages, and (iii) hemin, the ferric chloride salt of heme, increases the ferrous or ferric heme portion of the intracellular pool in these cells. Due to the reductive environment inside the cell, we expect the intracellular heme pool to contain iron predominantly in its ferrous form. Thus, we expected that treatment with hemin would result in discernable amounts of ferrous heme within J774A.1 cells. Our ESR analyses of heme-treated J774A.1 cells confirmed that a portion of heme exists in its ferrous form, since treatment with NO resulted in a clear nitrosylated heme ESR signal, which was absent in the cell supernatants. However, when cells were treated with NO throughout the incubation period, a stronger signal was determined, indicating an accumulation of the ferrous heme. This suggests that hemin treatment feeds the intracellular heme pool predominately with ferric heme. Our findings further indicate that NO may shift the balance between ferrous and the ferric heme toward the ferrous heme, thereby decreasing the portion of ferric heme. Upregulation of NOS under pro-inflammatory conditions yields higher levels of NO and is therefore supposed to favor the balance toward ferrous heme. Ferric heme, however, is the substrate of HO (50), which is supposed to predominantly decrease the portion of ferric heme. Thus, it is tempting to speculate that it is the balance between the ferrous and the ferric portion of the heme pool, which determines the predominant phenotype of the macrophage.

We admit as a limitation of our study that modulating effects of the essential enzymes NOS, HO, and NOX were obtained by indirect means, i.e., by using inhibitors of HO and NOS. Correspondingly, for impairing NOX activity, we used DPI, which is not a selective inhibitor of NADPH oxidases, but an inhibitor of all flavoproteins. For all inhibitors, we have chosen the lowest concentration that is possible to avoid excessive effects. However, further experiments are warranted to confirm our findings, ideally by using complementary methods.

## Conclusion

We showed that both enzymes NOS and HO are essential for important functions of macrophages, namely ROS generation and phagocytosis. While NOS, *via* its product NO, enhances ROS production, HO indirectly, *via* decreasing intracellular heme levels, enhances phagocytosis. This indicates that both enzymes contribute to the bactericidal activity of macrophages independently by controlling different pathways.

## Author Contributions

CD, AK, and AM were responsible for conception and design of the study. AM and GD performed experiments and analyzed data. CD and AK interpreted data and supervised the study. AM, CD, and AK wrote the manuscript. All authors read and approved the final manuscript.

## Conflict of Interest Statement

The authors declare that the research was conducted in the absence of any commercial or financial relationships that could be construed as a potential conflict of interest.

## Abbreviations

CO, carbon monoxide; COX, cytochrome oxidase; CrMP, chromium mesoporphyrin; DEA/NO, diethylamine NONOate; DETA/NO, diethylenetriamine NONOate; DPI, diphenyleneiodonium chloride; ESR, electron spin resonance; HO, heme oxygenase; HRP, horseradish peroxidase; l-NAME, l-N^G^-nitro-arginine methyl ester; mETC, mitochondrial electron transport chain; mtROS, mitochondrial ROS; NADPH, nicotinamide adenine dinucleotide phosphate; NO, nitric oxide; NOS, nitric oxide synthase; NOX, NADPH oxidase; NOX-ROS, NADPH oxidase-derived ROS; PMA, phorbol 12-myristate 13-acetate; RONS, reactive oxygen and nitrogen species; ROS, reactive oxygen species; SnPP, tin protoporphyrin; TC, *S*-methyl-l-thiocitrulline; ZnPP, zinc protoporphyrin.
